# Binary Particle Swarm Optimization Intelligent Feature Optimization Algorithm-Based Magnetic Resonance Image in the Diagnosis of Adrenal Tumor

**DOI:** 10.1155/2022/5143757

**Published:** 2022-02-28

**Authors:** Jian Xu, Fei Tian, Lei Wang, Zhongchang Miao

**Affiliations:** ^1^Department of Radiology, The First People's Hospital of Lianyungang, Lianyungang 222061, Jiangsu, China; ^2^Yingbo Super Computing (Nanjing) Technology Co. Ltd., Nanjing 210000, Jiangsu, China; ^3^Department of Radiation Oncology, The First People's Hospital of Lianyungang, Lianyungang 222061, Jiangsu, China

## Abstract

This research was aimed to explore the application value of magnetic resonance imaging (MRI) based on binary particle swarm optimization algorithm (BPSO) in the diagnosis of adrenal tumors. 120 patients with adrenal tumors admitted to the hospital were selected and randomly divided into the control group (conventional MRI examination) and the observation group (MRI examination based on the BPSO intelligent feature optimization algorithm), with 60 cases in each group. The sensitivity, specificity, accuracy, and Kappa of the diagnostic methods were compared between the two groups. The results showed that the calculation rate of the BPSO algorithm was the best under the same processing effect (*P* *<* 0.05). Optimization algorithm-based MRI is used in the diagnosis of adrenal tumors, and the results showed that the sensitivity, specificity, accuracy, and Kappa (83.33%, 79.17%, 81.67%, and 0.69) of the observation group were higher than those of the control group (50%, 75%, 58.33%, and 0.45). The similarity of tumor location results in the observation group (89.24%) was significantly higher than that in the control group (65.9%) (*P* *<* 0.05). In conclusion, compared with SFFS and other algorithms, the BPSO algorithm has more advantages in calculation speed. MRI based on the BPSO intelligent feature optimization algorithm has a good diagnostic effect and higher accuracy in adrenal tumors, showing the good development prospects of computer intelligence technology in the field of medicine.

## 1. Introduction

Adrenal tumor is a relatively common tumor disease in clinical practice. Adrenal tumor is located behind the peritoneum, the position is relatively fixed, and there is natural fat around, so it basically does not change with the movement of the abdomen and the pulsation of the great blood vessels [1, 2]. There are various classification methods for adrenal tumors, such as benign and malignant, existence of endocrine function, and location of tumors [3]. Patients with adrenal tumors can have symptoms such as hypertension, fatigue, blurred vision, thirst, irritability, hypokalemia, and water-electrolyte imbalance [4], which have a negative impact on the normal life of patients. Therefore, timely diagnosis and treatment of adrenal tumor patients is more important.

With the rapid development of medical technology, imaging has become the examination method for many diseases, which is widely used in the screening of diseases and the diagnosis of tumor diseases. At present, CT and magnetic resonance imaging (MRI) are commonly used in the clinical diagnosis of adrenal tumors [5]. The location of the adrenal gland and surrounding tissues create good conditions for CT examination. CT can be used to screen whether adrenal tumors exist, but it cannot determine whether they are benign or malignant. Multislice spiral CT-enhanced scan can observe the morphology, density, and intensity of the tumor to identify the benign and malignant adrenal tumors [6, 7]. However, in recent years, MRI has more clinical application value than CT. MRI scanning technology has the advantages of a series of examinations such as multidirectional, multiparameter, and multisequence and has a high resolution of soft tissue. It can show small lesions, and the surrounding blood vessels can be observed without the use of contrast agents. It is safer and more convenient, so it is widely recognized by clinicians [8, 9]. In particular, MRI in-phase inversion imaging technology, which is sensitive to the detection of lipid components in lesions, is helpful for the qualitative diagnosis of lesions. For abdominal examination, it has become a routine scanning sequence. In order to improve the accuracy of clinical examination and diagnosis, intelligent algorithms are widely used in medical image processing.

Nowadays, there are many image processing techniques for computer-aided diagnosis. The key to computer-aided diagnosis technology for medical image processing is the processing of image features, and the selection of image features is one of the most important links [10, 11]. Particle swarm optimization (PSO) is an evolutionary computation technology [12], which is derived from the research on the behavior of bird predation—a random search algorithm based on group collaboration developed by simulating the behavior of bird foraging. PSO can deal with some problems that cannot be dealt with by traditional methods, such as the lack of gradient information, but its performance is flawed, so someone proposed the binary PSO (BPSO) [13]. In the BPSO algorithm, each dimension of each particle is taken as a binary discrete value, namely, 0 or 1, and there is no limit to speed.

In order to further understand the application value of the BPSO algorithm in medical image processing technology, this study will use MRI technology based on the BPSO intelligent optimization algorithm to diagnose adrenal tumor patients and evaluate the diagnostic effect based on the results of surgical pathology as the gold standard, so as to provide more research basis for the clinical diagnosis of adrenal tumor patients and improve the cure rate of adrenal tumor patients.

## 2. Research Methods

### 2.1. Subjects

In this study, 120 patients with adrenal tumors admitted to the hospital from March 2019 to April 2021 were selected. Among them, 72 were male and 48 were female aged from 35 to 80 years, with an average age of (49.78 ± 4.32) years. A total of 52 patients with malignant tumors and 68 patients with benign tumors were preliminarily diagnosed clinically. The tumor location types of patients included metastatic tumors, stromal tumors, medullary tumors, and cortical tumors, including 27 patients with metastatic tumors, 35 patients with stromal tumors, 39 patients with medullary tumors, and 19 patients with cortical tumors. According to the random double-blind extraction method, all patients were divided into two groups, with 60 cases in each group. A group of patients with conventional MRI examination was set as the control group. A group of MRI imaging detection methods based on the BPSO intelligent feature optimization algorithm is set as the observation group. This study has been approved by the ethics committee of the hospital, and the family members of the patients were informed and signed the informed consent.

Inclusion criteria were defined as follows: (a) patients selected in this study all had single tumors, (b) the tumor diameter of all patients was above 0.5 cm, and (c) all patients are prepared for surgical treatment and pathological examination.

Exclusion criteria were defined as follows: (a) patients with cardiac pacemakers or metal foreign bodies and fear of seclusion are not suitable for MRI examination, (b) patients with tumor diameter less than 0.5 cm, and (c) patients with mental disorders.

### 2.2. BPSO Intelligent Feature Optimization Algorithm

#### 2.2.1. PSO Algorithm

The initialization state of PSO is a group of randomly combined particles. Assuming that a point in the S-dimensional space is a particle, then the *n*th particle can be expressed as *X*_*n*_={*x*_*n*_1__, *x*_*n*_2__,…, *x*_*n*_*s*__}, the best particle in each particle can be expressed as *P*_*n*_=(*p*_*n*_1__, *p*_*n*_2__,…, *p*_*n*_*s*__), and the best particle is set as gbest. The change rate of the *n*th particle can be expressed as *V*_*n*_=(*v*_*n*_1__, *v*_*n*_2__,…, *v*_*n*_*s*__). Particles can be changed according to the following equations:

(1) $$\begin{array}{c} w\left(t\right)=w_{\max}-\left(w_{\max}-w_{\min}\right),\\ X_{n_{i}}\left(t+1\right)=x_{n_{i}}\left(t\right)+v_{n_{i}}\left(t\right),\\ V_{n_{i}}\left(t+1\right)=w\left(t\right)*V_{n_{i}}+d_{1}*\text{rand}\left(\right)*\left(P_{n_{i}}\left(t\right)-x_{n_{i}}\left(t\right)\right)+d_{2}*\text{Rand}\left(\right)*\left(P_{g_{i}}\left(t\right)-x_{n_{i}}\left(t\right)\right). \end{array}$$

In the above two equations, *t* is the iteration times, *i*=1,2,…, S, *w* is the weight factor (a linear function that can change over time), and *d*_1_ and *d*_2_ are acceleration parameters (the attraction weights of gbest and the best particle to the particle). PSO algorithm is similar to the genetic algorithm, which is based on iterative optimization technology. The initialization of the system is a random combination, and the optimal results are searched based on an iterative algorithm. However, PSO searches according to the optimal particles in the discrete space, and there is no crossover and mutation in the genetic algorithm.

#### 2.2.2. BPSO Algorithm

The role of the BPSO algorithm is mainly to solve the optimization problem in discrete space. The particle position is represented as 0 and 1. The algorithm of binary particle swarm was first proposed by Kennedy and Eberhart [14]. According to the method of probability, the position of particles was updated by combining the algorithm with the speed. The Sigmoid function is used as the conversion function to map the particle position to 0 or 1 in the binary encoding.

(2) $$\begin{array}{c} x_{n_{i}}=\left\{\begin{array}{cc} 1, & \text{rand}\left(\right)<S\left(v_{n_{i}}\right)\\ 0, & \text{otherwise} \end{array}\right.,\\ S\left(v_{n_{i}}\right)=\frac{1}{1+e^{-V_{n_{i}}}}. \end{array}$$

In the above two equations, *v*_*n*_*i*__ and *x*_*n*_*i*__ take the probability of 1,0 rand() is the random number between 0 and 1.

#### 2.2.3. Update Strategy of BPSO Algorithm

Since the combination of feature subsets of the BPSO algorithm is a binary space, the speed change method in the traditional PSO algorithm cannot be applied. Therefore, the three indicators will be adjusted in the application process of the BPSO algorithm used in this study.

First, the parameters *m*1 and *m*2 will decrease with the increase of iterations. The range of *m*1 and *m*2 is as follows:

(3) $$\begin{array}{c} m2,\;\left(0<=\text{m}2<S\right),\\ m1,\;\left(0<=\text{m}1<S\right). \end{array}$$

The change equation is as follows:

(4) $$\begin{array}{c} m2\left(t\right)=\text{m}2*\frac{\left(100-t\right)}{100},\\ m1\left(t\right)=\text{m}1*\frac{\left(100-t\right)}{100}. \end{array}$$

The particle update is as follows: it is assumed that the number of iterations is *L*.

(a) For the best particle, the corresponding particle Ptmp can be obtained by randomly changing m1*∗*rand().
(b) For gbest, the corresponding particle Gtmp can be obtained by randomly changing m2*∗*rand().
(c) Then, the two particle values are compared; if they are the same, the same position is taken. If they are different, then the calculation is performed according to the coefficient, and the specific equation is as follows:
  (5) $$\begin{array}{c} \text{Bit}\left(c\right)=\left[{\text{bit}}_{Ptmp}+\left({\text{bit}}_{Gtmp}-{\text{bit}}_{Ptmp}\right)*\left(\text{rand}<l3\right)\right]. \end{array}$$

**Figure 1:**
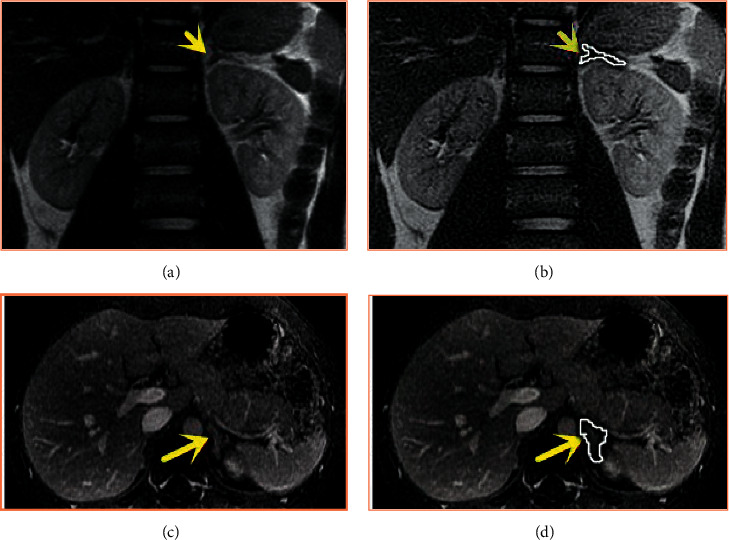
Image processing. (a, b) MRI images of different sequences before processing. (c, d) MRI images processed by BPSO algorithm. The yellow arrow refers to the location of the tumor.

The above equations are obtained by rounding. In steps (a) and (b), the random changes of pbest and gbest can make the motion direction of particles random. However, with the decrease of time and the sum of parameters, the above two changes will become smaller and smaller. In addition, step (c) enables the particles to approach the best particle and gbest simultaneously. In this study, the characteristics of MRI are extracted and segmented under this technology, and the processing effect is shown in Figure 1.

### 2.3. MRI Examination Method

The MRI examination of all patients was performed by the same imaging physician and was performed on the same MRI examination instrument. The MRI examination details are as follows.

All patients were scanned by 3.0 T MRI. The patients were trained to breathe and breath-hold scanning. First, conventional sequence scanning was performed, including axial and coronal scanning. Secondly, cross-sectional chemical displacement imaging sequence scanning was performed. Finally, diffusion-weighted imaging (DWI) sequence scanning was performed.  Table 1 shows specific parameters.

### 2.4. Observation Indicators

The size of adrenal tumors in the two groups was compared and analyzed. By comparing with the results of the pathological examination, the diagnostic sensitivity, specificity, accuracy, and consistency of the two groups of patients in the determination of the location and nature of adrenal tumors were analyzed.

### 2.5. Statistical Methods

The SPSS22.0 statistical software system was used for data entry, collation, and statistical analysis. *χ*^2^ test was used for comparison of count data. Measurement data were expressed by *t*-test. Variance analysis was used to compare the mean of multiple samples. LSD method was used when the variance was uniform, and the Dunnett T3 method was used when the variance was not uniform. *P* *<* 0.05 was statistically significant. Kappa test was performed for the consistency between the two groups of examination results and the results of surgical pathology. When Kappa >0.75, the consistency was strong, and when 0.4 ≤ Kappa <0.75, the consistency was general. When Kappa <0.4, the consistency was poor.

## 3. Results

### 3.1. Effect Comparison of the BPSO Algorithm

**Figure 2:**
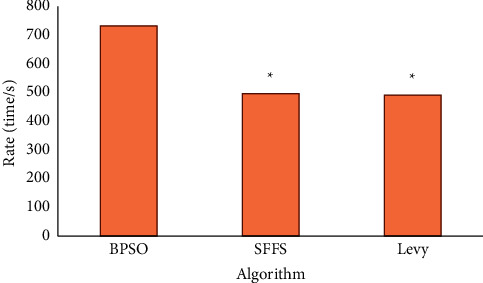
Comparison of algorithm speed. Note: “^*∗*^” indicates that the difference was statistically significant (*P* < 0.05).

**Figure 3:**
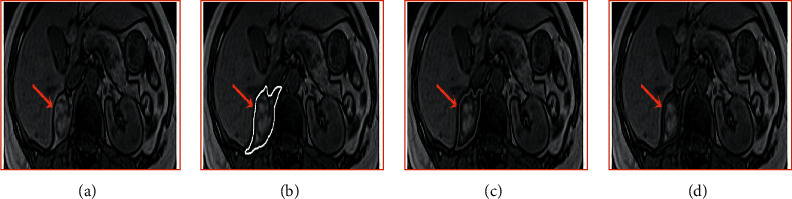
Comparison of image processing results. (a) Unprocessed image. (b) BPSO. (c) SFFS. (d) Levy (red arrow indicates the location of the tumor).

Figure 2 shows the comparison of the algorithm speed between the BPSO algorithm and other algorithms (sequential floating forward selection (SFFS) and the Levy algorithm). According to the results of the graph, the calculation speed of the BPSO algorithm (730 times/s) is significantly higher than that of the other two algorithms (SFFS, 495 times/s; Levy, 490 times/s) (*P* *<* 0.05), which indicates that the BPSO algorithm is better under the same processing effect.  Figure 3 shows the comparison of the processing effect of the three algorithms. The BPSO algorithm is more obvious in the segmentation and extraction of image features.

### 3.2. Comparison of General Data between the Two Groups of Patients

**Figure 4:**
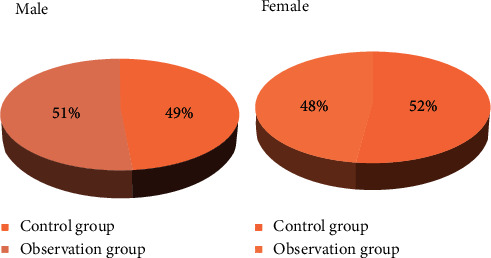
Gender distribution.

**Figure 5:**
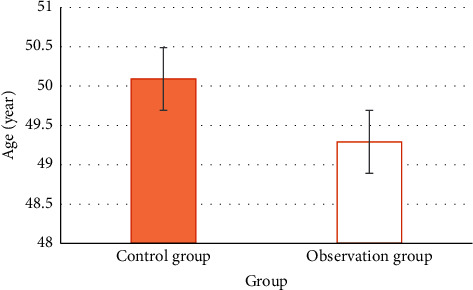
Average age comparison.

**Figure 6:**
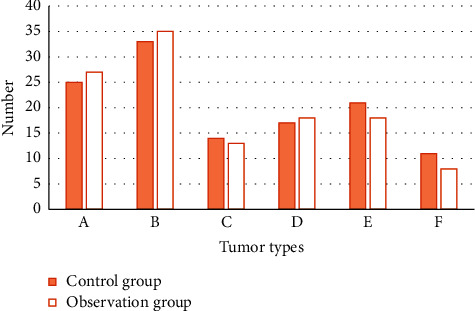
Distribution of tumor types. (a) Malignant tumor. (b) Benign tumor. (c) Metastases. (d) Stromal tumor. (e) Myeloma. (f) Cortical tumor.

Figure 4 shows the gender distribution of the two groups of patients, including the control group, 35 male patients (48.61%) and 25 female patients (52.08%), and the observation group, 37 male patients (51.39%) and 23 female patients (47.92%).The comparison was not statistically significant (*P* *>* 0.05).  Figure 5 shows the comparison of the average age of the two groups. The average age of the control group was (50.09 ± 4.51) years. The average age of patients in the observation group was (49.29 ± 4.21) years, and there was no significant difference (*P* *>* 0.05).  Figure 6 shows the distribution of tumor types. In the control group, there were 25 patients with malignant tumors, 33 patients with benign tumors, 14 patients with metastatic tumors, 17 patients with stromal tumors, 21 patients with medullary tumors, and 11 patients with cortical tumors. There were 27 patients with malignant tumors, 35 patients with benign tumors, 13 patients with metastatic tumors, 18 patients with stromal tumors, 18 patients with medullary tumors, and 8 patients with cortical tumors in the observation group. There was no significant statistical significance (*P* *>* 0.05).

### 3.3. MRI Results of Different Types of Tumors

**Figure 7:**
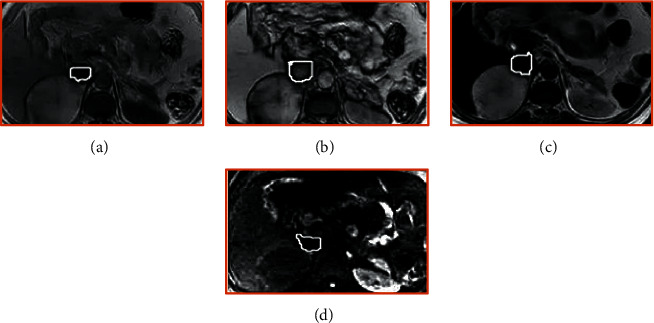
Adrenal stromal tumors. (a) T1WI in-phase. (b) T1WI antiphase. (c) T2WI. (d) DWI.

**Figure 8:**
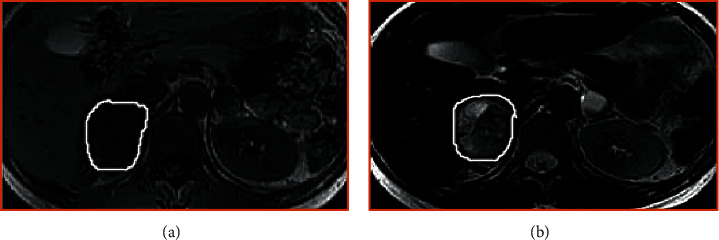
Adrenal medullary tumor. (a) T1WI. (b) T2WI.

**Figure 9:**
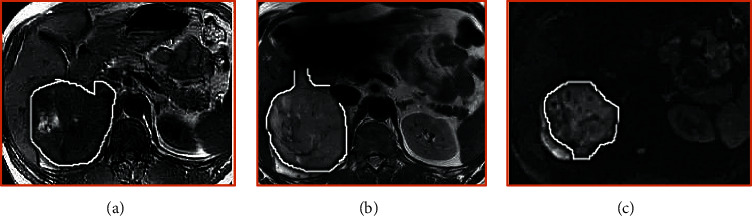
Adrenocortical tumors. (a) T1WI. (b) T2WI. (c) DWI.

The MRI imaging results of different types of tumors are as follows: the right adrenal round tumor in patients with adrenal stromal tumors has clear boundaries. The positive and negative phases of T1WI and T2WI are high signals. The signal intensity of the T2WI fat suppression sequence is unevenly reduced. DWI shows that the tumor has uniform low signal changes (Figure 7). The adrenal lobulated tumors in patients with adrenal medullary tumors had clear boundaries and low signal intensity on T1WI. On T2WI, medium and high signal intensities were dominant, and a high signal cystic area was observed at the edge. The cystic and necrotic areas showed small, round, and obvious high signal intensity shadow, and the signal distribution on the enhanced scan was uneven, as shown in Figure 8. This circular, lobulated, ill-defined adrenal mass in patients with adrenal cortical tumor was characterized by low signal intensity on T1WI and patchy areas of high signal intensity (bleeding) and mixed signal changes with slightly high signal intensity on T2WI. DWI showed that the tumor was diffusively limited and showed uneven high signal (Figure 9).

### 3.4. Comparison of Diagnostic Accuracy of Tumor Nature

**Figure 10:**
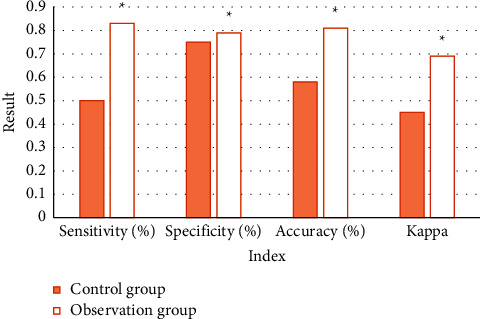
Comparison of diagnostic effects. Note: “^*∗*^” indicates that the comparison was statistically significant (*P* < 0.05).

Table 2 shows the statistics of the diagnostic results of tumor properties under MRI and pathological examination in the control group. According to the calculation, the sensitivity, specificity, and accuracy of the diagnostic results obtained in the control group were 50%, 75%, and 58.33%, and the Kappa value was 0.45, respectively.  Table 3 shows the statistics of the diagnostic results of tumor properties under MRI and pathological examination in the observation group. According to the calculation, the sensitivity, specificity, and accuracy of the diagnostic results obtained in the observation group were 83.33%, 79.17%, and 81.67%, respectively, and the Kappa value was 0.69. The sensitivity, specificity, accuracy, and Kappa of the diagnosis results in the observation group were higher than those in the control group (*P* *<* 0.05) (Figure 10).

### 3.5. Comparison of Tumor Location Diagnosis

**Figure 11:**
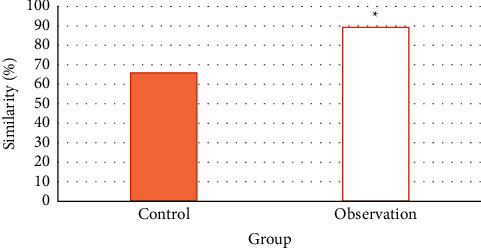
Similarity comparison. Note: “^*∗*^” indicates that the comparison was statistically significant (*P* < 0.05).

Table 4 shows the statistics of the results of MRI examination and surgical pathology in the tumor location of the two groups. According to the statistical results, the average similarity between the MRI examination results and the pathological results of the control group is 65.9%, and the average similarity of the observation group is 89.24%. The similarity of the examination results of the observation group is significantly higher than that of the control group (*P* *<* 0.05) (Figure 11).

## 4. Discussion

Adrenal gland is a kind of gland located on the human kidney, which is consistent with the number of kidneys. It is an important endocrine organ in the human body. Because of their close relationship with the kidney, adrenal tumors are classified as urological diseases. There are many examination methods for adrenal tumors. In this study, MRI technology was used as the main research technology, combined with the image processing technology in the widely developed computer-aided diagnosis technology, and the application value of MRI was evaluated.

PSO calculation and binary algorithm results formed the BPSO algorithm, which is used as the processing technology of MRI image of adrenal tumor patients in this study. This technology can enhance the image features and segment the image to increase the accuracy of the examination results. The results show that the BPSO algorithm can effectively identify and extract the characteristics of clinical data [15]. In this study, the BPSO algorithm is compared with the SFFS algorithm and the Levy algorithm. The results show that, under the same processing effect, the calculation rate of the BPSO algorithm is the best, suggesting the feasibility of the BPSO algorithm in this study. Xiong et al. [16] also pointed out in a study on efficient gene selection methods for microarray data based on LASSO and BPSO that the improved BPSO can select the best gene subset with high probability by using the compact gene library obtained by double filtering strategy. Compared with the related methods, the experimental results on several common microarray data using limit learning machines verify the effectiveness of the proposed method. Another study on multigroup heterogeneous binary PSO using the win-win method to improve feature selection in liver and kidney disease diagnosis shows that, with the help of this algorithm, the relevant system classifies tumors as benign or malignant with the minimum error rate [17]. This is basically consistent with the results of this study, which shows that people can improve the accuracy of benign and malignant tumor diagnosis with the support of the BPSO algorithm. In this study, the sensitivity, specificity, accuracy, and Kappa (83.33%, 79.17%, 81.67%, and 0.69) of the diagnosis results of the observation group are higher than those of the control group (50%, 75%, 58.33%, and 0.45). The similarity between the examination results of tumor location and pathological examination results in the observation group (89.24%) was significantly higher than that in the control group (65.9%). All these suggest that the BPSO algorithm has good value in image processing. Of course, it is not only used for MRI image processing. Research shows that the BPSO algorithm can also be applied to EMG processing [18], optimization of distance deviation naive Bayesian classification method [19], and performance optimization processing of other algorithms [20–22], showing that the algorithm has a wide range of application fields.

## 5. Conclusion

The BPSO algorithm, formed of PSO calculation and binary algorithm results as the processing technology of MRI images of adrenal tumor patients, was used to evaluate the application value of MRI based on the BPSO intelligent feature optimization algorithm in adrenal tumor diagnosis. In conclusion, the BPSO intelligent feature optimization algorithm is more effective in image processing; MRI based on this algorithm has obvious advantages in the diagnosis of adrenal tumors, with good accuracy, which has good clinical application value. However, the comparative indicators are insufficient, and the relationship between disease location and benign and malignant tumors is not studied, which will be improved in future research. It shows the good development prospects of computer intelligence technology in the medical field.

## Figures and Tables

**Table 1:** DWI scan parameters.

Scanning sequence		Time of repetition (TR) (ms)	Time of echo (TE) (ms)
Conventional sequence	Coronal scanning T2WI	1400	92
	Cross-sectional scanning T2WI	3000	103
Chemical displacement imaging sequence	In phase	130	2.30
	Antiphase	130	3.70
Diffusion-weighted imaging		3936	75

**Table 2:** Statistics of the diagnosis results of tumor properties in the control group.

Inspection method		Pathological results		Total
		Malignant	Benign	
MRI	Malignant	20	5	25
	Benign	20	15	35
Total		40	20	60

**Table 3:** Statistics of the diagnosis results of tumor properties in the observation group.

Inspection method		Pathological results		Total
		Malignant	Benign	
MRI	Malignant	30	5	35
	Benign	6	19	25
Total		36	24	60

**Table 4:** Statistical results of tumor location diagnosis.

Tumor type	Control group (*n* = 60)		Observation group (*n* = 60)	
	MRI	Pathological result	MRI	Pathological result
Metastatic tumor	14	19	17	18
Stromal tumor	17	23	20	20
Medullary tumor	21	11	14	16
Cortical tumor	11	7	8	6

## Data Availability

The data used to support the findings of this study are available from the corresponding author upon request.

## References

[1] Almeida M., Bezerra-Neto J., Mendonça B., Latronico A., Fragoso M. (2018). Primary malignant tumors of the adrenal glands. *Clinics*.

[2] Shah M. H., Goldner W. S., Halfdanarson T. R. (2018). NCCN guidelines insights: neuroendocrine and adrenal tumors, version 2.2018. *Journal of the National Comprehensive Cancer Network*.

[3] Crona J., Beuschlein F., Pacak K., Skogseid B. (2018). Advances in adrenal tumors 2018. *Endocrine-Related Cancer*.

[4] Lam A. K.-y. (2017). Update on adrenal tumours in 2017 world health organization (WHO) of endocrine tumours. *Endocrine Pathology*.

[5] Bhargava P., Sangster G., Haque K., Garrett J., Donato M., D’Agostino H. (2019). A multimodality review of adrenal tumors. *Current Problems in Diagnostic Radiology*.

[6] Frey S., Caillard C., Toulgoat F., Drui D., Hamy A., Mirallié É. (2020). Non-adrenal tumors of the adrenal area; what are the pitfalls?. *Journal of Visceral Surgery*.

[7] Wong K. K., Chondrogiannis S., Fuster D. (2017). Additional value of hybrid SPECT/CT systems in neuroendocrine tumors, adrenal tumors, pheochromocytomas and paragangliomas. *Revista Española de Medicina Nuclear e Imagen Molecular*.

[8] Elsayes K. M., Elmohr M. M., Javadi S. (2020). Mimics, pitfalls, and misdiagnoses of adrenal masses on CT and MRI. *Abdominal Radiology*.

[9] Aggarwal A., Das C. J. (2021). Contrast-enhanced ultrasound in evaluation of adrenal lesions with CT/MRI correlation. *British Journal of Radiology*.

[10] Lv Z. H., Chen D. L., Cao B., Song H. B., Lv H. B. (2021). Secure deep learning in defense in deep-learning-as-a-service computing systems in digital twins. *IEEE Transactions on Computers*.

[11] Lv Z. H., Qiao L., Wang Q. J., Piccialli F. (2020). Advanced machine-learning methods for brain-computer interfacing. *IEEE/ACM Transactions on Computational Biology and Bioinformatics*.

[12] Guo S. S., Wang J. S., Guo M. W. (2020). Z-shaped transfer functions for binary particle swarm optimization algorithm. *Computational Intelligence and Neuroscience*.

[13] Nguyen B. H., Xue B., Andreae P., Zhang M. (2021). A new binary particle swarm optimization approach: momentum and dynamic balance between exploration and exploitation. *IEEE Transactions on Cybernetics*.

[14] Jahandideh-Tehrani M., Bozorg-Haddad O., Loáiciga H. A. (2020). Application of particle swarm optimization to water management: an introduction and overview. *Environmental Monitoring and Assessment*.

[15] Pashaei E., Pashaei E., Aydin N. (2019). Gene selection using hybrid binary black hole algorithm and modified binary particle swarm optimization. *Genomics*.

[16] Xiong Y., Ling Q. H., Han F., Liu Q. H. (2019). An efficient gene selection method for microarray data based on LASSO and BPSO. *BMC Bioinformatics*.

[17] Gunasundari S., Janakiraman S., Meenambal S. (2018). Multiswarm heterogeneous binary PSO using win-win approach for improved feature selection in liver and kidney disease diagnosis. *Computerized Medical Imaging and Graphics*.

[18] Karthick P. A., Ghosh D. M., Ramakrishnan S. (2018). Surface electromyography based muscle fatigue detection using high-resolution time-frequency methods and machine learning algorithms. *Computer Methods and Programs in Biomedicine*.

[19] Shaban W. M., Rabie A. H., Saleh A. I., Abo-Elsoud M. A. (2021). Accurate detection of COVID-19 patients based on distance biased Naïve Bayes (DBNB) classification strategy. *Pattern Recognition*.

[20] Li H., Zhang L. (2021). A bilevel learning model and algorithm for self-organizing feed-forward neural networks for pattern classification. *IEEE Transactions on Neural Networks and Learning Systems*.

[21] Huang X., Zeng X., Han R. (2017). Dynamic inertia weight binary bat algorithm with neighborhood search. *Computational Intelligence and Neuroscience*.

[22] Sun T., Xu M.-h. (2017). A swarm optimization genetic algorithm based on quantum-behaved particle swarm optimization. *Computational Intelligence and Neuroscience*.

